# The Metropolitan Hospital Sunday Fund

**Published:** 1895-06-29

**Authors:** 


### The Metropolitan Hospital Sunday Fund.

### FIRST LIST OF DONATIONS.

Donations and cheques continue to be sent to the Lord Mayor or the Secretary at the Mansion House. Admiral Ward and others have promised to double their contributions, if necessary, in order to raise the total contributions to £70,000 this year. The following is the first list of donations from the 14th to the 24th inst. Upwards of £30,000 had been paid in already.

In former years individual gifts of from £5,000 to £1,000 have been received. It is hoped that several may emulate Mr. Robinson's generosity by giving £1,000 each before the Fund closes this year.

**Donation of £1,000.**

J. B. Robinson.

**Donations of £105.**

Lion Brewery Company (Lim.).
G. Palmer.

**Donations of £100.**

C. W. Rudd.
Earl of Feversham (a further).
Arthur James.

**Donation of £50.**

J. G. A. Baird, M.P.

**Donation of £35.**

Miss E. Wright.

**Donation of £31 10s.**

Marks, Bulteel, Mills, and Co.

**Donations of £25.**

F. Druce (a further).
A. Milln.
Miss Emily Old (a further).
J. J. Randolph (a further).

**Donations of £21.**

H. Trower.
E. Heseltine.
C. H. T. Hawkins.

**Donations of £20.**

W. M. Cross, C.C.
Major Jones (a further).
W. T. Blanford.
Lord Hindlip.
Mrs. Burnaby (a further).

**Donations of £10 10s.**

W. D. Graham and Menzies.
Theophilus Sandeman.
Sir Henry S. Rawlinson.
Young, Jones, and Co.
T. Gooch and Sons.
Gosling and Sharpe (a further).

**Donations of £10.**

John Young.
R. B. Martin, M.P. (a further).
W. D. James.
Captain J. A. Orr Ewing.
A. F. Williams.
B. M. Woollan.
Mrs. Pandely Mavrogordato.
Mrs. Kennedy (a further).
The Countess of Cork.
Arthur G. Phillips.
Archibald Bulman (a further).
Edward L. Benyon.

**Donation of £6 3s. 6d.**

The Assistants of Mr. E. A. Barker, King's Road, Chelsea.

**Donations of £5 5s.**

A. S. Churchill.
G. A. Macmillan.
D. C. S. L. Carnegie (a further).
E. D. and F. Man (a further).
Miss Sneyd (a further).
F. R. Whalley (a further).
Charles Gold, jun.
A. D. Gordon (a further).

**Donations of £5.**

Mrs. Webb Edge
Mrs. Lumsden.
H. G. Cattley.
Geo. F. Smart.
Adolph B. H. Goldschmidt.
Mrs. J. W. Smith.
Baron Von Olegar.
H. Richardson.
Robert Parker (a further).
Edwin Hall (a further).
Miss Russell.
James Johnston (a further).
Heath Harrison.
Miss Emma C. Chamberlayne.
Mrs. Anne Place.
C. H. Moore.
Miss L. M. Van der Weyer.
Miss Eley.
G. F. Pocock.
Miss Phillips.
Vice-Admiral A. Courteney (a further).
Mrs. Longdon (a further).
David Longdon (a further).
Joseph Ruston.
C. E. Richardson.
J. C. Salt (a further).
Miss C. J. Hartridge.
W. B. Gair.
Lord F. FitzRoy.
Miss Church.
J. Smith.
A. E. F. Horniman.
Mrs. M. A. French.
Mrs. Rivington (a further).
Mrs. Henry Bentnich (a further).
R. L'Amy (a further).
Misses Rawson.
Henry Furmor.
Mrs. Roland Whitehead.
Miss Meade.
Robert Gordon.
Miss Gregory.
W. Freke Evans.
Lieut.-Colonel Boyd Alexander.
F. C. Anderson.

**Donations of £4.**

John Annan.
J. Grugeon.

**Donations of £3 3s.**

Johnson Keeller.
Mrs. Hales Wilkie.
Lord Knutsford (a further).
Allan Lambert.
Walter Jacobs.
T. Hugh Lee.

**Donations of £3.**

Major-General H. Hammond.
Mrs. William Penn.
S. B. Bristowe.
A. Boyd.

**Donation of £2 12s. 6d.**

Mrs. Lucy Russell.

**Donations of £2 2s.**

Edward H. Rea, C.M.G.
L. F. Topham.
Spreckley, White, and Lewis.
R. Hardie.
Bolton, Piltand, and Breden.
Chaplin and Co.
Lucas and Spencer's Wharf (Lim.)
W. G. Gould.
J. T. Bolding.
Rev. J. W. Parsons.
R. C. Allen.
W. Rigby,
S. G. Holland.
W. A. Satchell, M.D. (a further)
Misses Du Bois.
J. Cahn.
G. Borthwick (a further).
E. P. Williamson.
W. H. Fricker (a further).
J. P. Williams (a further).
Mrs. Jenkins Jones.
Mrs. Ackenhead.
Robert Porter.
Sir Joseph Fayrer.
W. A. Geare.
Mr. Deputy Creasey.
J. J. Sylvester.
F. K. North (a further).
J. Roberts.

**Donations of £2.**

W, Heald.
Mr. and Mrs. W. J. Reade.
Peter Williams.
J. Hudson.
J. B. McIntyre.
R. Paul (a further).
Miss Hall.
Harrison Watson.
Misses Lees.
R. G. Buist.
Mrs. Kays.
Mrs. Fulton.
Colonel J. M. McNeile, R.E.
A. Liddell.
Mrs. Bullen Smith.

**Donations of £1 10s**

Robert Frost.
Hon. Maude Lawrence.
Miss Trimmer.

**Donation of £1 4s. 4d.**

G. W. Bell and family.

**Donations of £1 1s.**

General Schneider.
H. L. Bolton.
W. J. Hankin.
D. Jay.
Mrs. Gilmore.
P. T. Ogle.
E. Money Wigram.
C. Ridley Smith.
James Hart.
F. Filliter (a further).
Mrs. W. S. Halsey (a further).
W. Mortimer.
B. Lester.
J. A. F. Gregg.
E. B. Sutton.
K. R. Fletcher.
J. Fallowfield.
E. H. Anson.
W. T. Smith.
B. D. Jackson.
G. Cloutte.
H. J. Bound.
General T. H. Donne.
Arthur Hughes.
W. J. Beard.
F. Hess.
Mrs. Edward Smith.
Hugh Forrester.
Miss Rose (a further).
F. Dutton.
Rev. John Jackson, M.A.
G. Greiner.
J. G. S. Anderson.
Mrs. Anderson.
H. M. Pollett.
Mrs. W. C. Fairley.
W. White.
Mrs. Yates (a further).
A. Webster (a further).
E. Knox.
J. Plummer (a further).
Mrs. Bolland Newton (a further).
T. W. Garlike.
Miss A. E. Griffiths.
A. Harvey.
W. G. Fossick.

**Donations of £1.**

J. B. Lubbock.
General A. Perkins.
Miss L. Palmer.
Lady Henrietta Pelham.
The Spanish Ambassador.
H. Oppenheimer.
E. J. W. Gibb.
General Duncan.
Leigh Heseltine.
H. W. Henderson.
Miss Magnay.
Dean Lake.
S. C. Rider.
Duchess of Wellington.
W. Chapman (a further).
W. Coombes (a further).
T. Scovell.
Miss Drummond.
W. G. Devon Astle.
E. C. Floyd.
W. H. Brown.
Mrs. Langdale.
E. R. Hughes.
Lieut.-Col. Gerald Martin.
Nath. Cohen.
A. T. Binny.
Hon. Mrs. Jervoise Smith.
Mrs. Hailstone.
Miss Mary Arding.
B. Depledge.
R. C. Coode.
Miss Coode.
Miss W. A. Coode.
Mrs. Peter Redpath.
J. B. Ritchie.
Mrs. Brookman.
W. H. Johnston.
C. H. Lomax (a further).
C. F. Inskipp.
T. M. Whitehead.
F. Hirtzel.
Lieut.-General Sir C. V. Tauner.
Mrs. Wright.
Rev. T. O'Beernan.
General H. Hopkinson.
Major-General H. P. Sykes.
Mrs. R. H. Bush.
F. A. Price.
Rev. M. Terry.
E. Walford.
Mrs. F. H. Maltby.
Miss B. D. Lewis.
Mrs. Clementi.
Miss Pigeon.
E. Trotter.
F. G. Reynolds.
H. Matthews.
W. B. Pattisson.
Miss E. P. Story.
Mrs. Russell.
Miss Annie S. Lawrie.
J. Brunskill.
Mrs. Oliver.
Captain R. E. Lambert.
H. J. Mills.
Mrs. Wood (Freeland).
H. G. J. Comb.

Donations under £1 and anonymous gifts amount to about £100. It is hoped that the donations may reach at least £10,000 this year, and the Lord Mayor will be glad to receive at the Mansion House many further cheques and contributions for large and small amounts.

